# Prevalence and Risk Factors of Gastroesophageal Reflux Among Jazan University Students, Saudi Arabia: A Cross-Sectional Study

**DOI:** 10.7759/cureus.22500

**Published:** 2022-02-22

**Authors:** Bander Otayf, Fatimah Dallak, Abeer Alomaish, Almuhannad Qadri, Reema Moafa, Ibrahim Gosadi, Abdulaziz H Alhazmi

**Affiliations:** 1 Medicine, Jazan University, Jazan, SAU; 2 Family and Community Medicine, Jazan University, Jazan, SAU; 3 Microbiology and Parasitology, Jazan University, Jazan, SAU

**Keywords:** saudi arabia, jazan, university students, risk factor, gerd, gastroesophageal reflux

## Abstract

Introduction: Gastroesophageal reflux disease (GERD) is a digestive disorder that is commonly seen in adults and characterized by heartburn and regurgitation. The epidemiological aspects of GERD have been studied over the past decade due to the increasing prevalence and complications of the disease. Thus, we aimed in this study to assess the prevalence, risk factors of GERD, and its relationship with academic performance among students at Jazan University.

Methods: This is a descriptive and analytical cross-sectional study that was conducted among Jazan university students in Jazan Province, Saudi Arabia. Data was collected using a self-administered questionnaire and analysis was performed using SPSS (IBM Corp., Armonk, NY).

Results: A total of 953 students participated in this study. The prevalence of GERD was found to be 23.1%. Our findings showed that five or more physical activities for ≥ 30 minutes per week, and fiber-rich foods were found to decrease the odds of developing GERD. However, more than three meals per day and having a family history of GERD were found to increase the odds of GERD. Moreover, age, use of proton pump inhibitors (PPI), and Khat chewing were also statistically significant risk factors for GERD (p<0.05).

Conclusion: Our results demonstrated a high prevalence of GERD among Jazan university students. However, risk factors were almost similar to those reported by previous studies. Thus, raising awareness about the modifiable risk factors of GERD is warranted.

## Introduction

Gastroesophageal reflux disease (GERD) is a condition characterized by uncomfortable symptoms including heartburn and/or acid regurgitation, which may be associated with an esophageal mucosal injury [1,2]. Some extra-esophageal symptoms could be experienced by affected patients, such as respiratory manifestations or sleep disturbance [3]. GERD can be chronic and may lead to ulcer formation in the esophagus, esophagitis, and numerous other complications [4]. Many risk factors have been associated with GERD, including consumption of alcohol or analgesics, family history, nutritional factors, high body mass index (BMI), lack of physical activities, and cigarette smoking. These risk factors may affect the lifestyle of the affected individual [5]. Over the past decade, several studies have reported a surge in the prevalence of GERD-related symptoms and complications [6].

The prevalence of GERD is varied globally, which is estimated to be 23% in South America, 18% to 28% in North America, 12% in Australia, 2% to 7% in East Asia, 9% to 26% in Europe, and 9% to 33% in the Middle East [1,2,7]. The evidence suggests that the prevalence of GERD has significantly increased since 1995, especially in East Asia and North America [7]. The prevalence of GERD in Saudi Arabia varied from 29% to 45% [2]. In the Saudi population, the prevalence of GERD is substantially higher compared to western countries and East Asian countries [2,8].

While GERD is a significant problem that can negatively affect the quality of life as well as daily activities [1,7], there are relatively few studies about the prevalence and impact of GERD among university students and how that could affect their quality of life, including academic performance. Thus, we aim to assess the prevalence and risk factors of GERD among students at Jazan University.

## Materials and methods

Study settings

The study is a descriptive and analytical cross-sectional study that was conducted among Jazan university students in the Jazan region, Saudi Arabia. Data collection was started on March 2021 with a duration of three weeks. We used a non-random, convenient sampling technique to choose our sample of university students among the whole population. Nonetheless, an effort was made to approach students from all faculties and both genders via the Deanship of Students Affairs which facilitated distributing the data collection tool to all university students.

Sample size

A sample of 372 participants is estimated to be necessary for this study, the sample size is calculated using the StatCal function of EpiInfo assuming the prevalence of GERD is 41% [1] with a 5% margin of error and 95% confidence level.

Data collection tools

Data was collected using a self-administered pretested questionnaire which was developed in the Arabic language [1]. The questionnaire consists of 36 questions and the questionnaires were distributed to Jazan university students. The questionnaire is divided into four sections: social and demographic data, risk factors and lifestyle, GerdQ for the diagnosis of GERD as well as complications. Twenty questionnaires were administered during a pilot study and the questionnaire was modified according to its results. The results of the pilot study were not included in the results. The scoring of GerdQ depends on the frequency of these symptoms during the last week (less than once, once, 2-3 times, and 4-7 times, respectively), where the scores range from 0 to 3 for the positive GERD predictors.

Data presentation & statistical analysis

We analyzed the data as follows: First, we described the dataset of the number, frequencies, percentages, and standard error of the sample of student variables in the study. Missing data and variables were removed as well as other variables not related to the study. Second, we tested each of the two dependent variables separately against each of the other predictor variables through using bivariate analysis or crosstabs procedure. Third, we used multivariate regression to analyze the data. Alongside using the multiple regression model to study the relationship, odds ratio, P-value, and 95% confidence intervals (CIs) were obtained. The data was collected, cleaned, and analyzed through SPSS v23.0 (IBM Corp., Armonk, NY). All tests were two-sided and a p-value <0.05 was considered statistically significant.

Ethical considerations

Jazan students were given informed consent to participate in data collection. The importance and benefits of the current study were explained. Participation was voluntary and verbal consent was acquired from all participants. Confidentiality of all participants was maintained as no names were mentioned in the questionnaires. Data collected from study participants were only used for scientific purposes. All the participants had the right to stay or withdraw from the study at any time. Ethical approval was obtained from the Institute Review Board (IRB) of Jazan University (REC/42/1/106), May 2, 2021.

## Results

Socio-demographic characteristics of the participants

A total of 953 participants were included in this study; most of them (59.7%) were female and 40.3% were male. The average age of the participants was 22 ± 2.9 years (range 17 - 54 years). The majority of the participants were single (86.5%) while the marital status for others was: married (12.5%), divorced (0.9%), and widowed (0.1%). Most of the participants (66.1%) lived in rural areas. As for the BMI of the participants, the average was 23 ± 5.4 (range 9.26 - 69.86) and most of them (52.6%) had a normal BMI.

About 28.8% of the participants were in the 4th year in university and more than half of them (53.5%) studying in non-health faculties. Most of the participants were students in the business administration faculty (13%) and faculty of medicine (13.6%). Regarding their academic performance, the average grade point average (GPA) was 4.1 ± 0.65 (range 1 - 5).

The relationship between GERD and different socio-demographic factors was assessed. A statistically significant association (p < 0.05) was found between age and GERD. The prevalence of GERD was higher among older participants. Other sociodemographic factors did not significantly associate with GERD. The characteristics of the participants are presented in Table 1.

**Table 1:** Sociodemographic characteristics of the participants and its association to GERD (n=953) *P values were calculated using chi-square test for categorical variables and independent samples T-test for continuous variables. Percentages were calculated within each row. GERD: gastroesophageal reflux disease; SD: standard deviation. GPA: grade point average. BMI: body mass index.

Variable	Category	GERD		Total N (%)	P-value
		Yes	No		
Age (Years): Mean (SD)		22.4 (2.86)	21.8 (3.12)	22 ± 2.9	0.018*
GPA: Mean (SD)		4.0 (0.68)	4.1 (0.64)	4.1 ± 0.65	0.255
Gender: N (%)	Male	87 (22.7)	297 (77.3)	384 (40.3)	0.796
	Female	133 (23.4)	436 (76.6)	569 (59.7)	
Marital status: N (%)	Single	182 (22.1)	642 (77.9)	824 (86.5)	0.056
	Married	33 (27.7)	86 (72.3)	119 (12.5)	
	Divorced	5 (55.6)	4 (44.4)	9 (0.9)	
	Widowed	0 (0)	1 (100)	1 (0.1)	
Residence: N (%)	Urban	76 (23.5)	247 (76.5)	323 (33.9)	0.816
	Rural	144 (22.9)	486 (77.1)	630 (66.1)	
BMI: N (%)	Underweight	47 (25.5)	137 (74.5)	184 (19.3)	0.698
	Healthy weight	112 (22.4)	389 (77.6)	501 (52.6)	
	Overweight	44 (24.2)	138 (75.8)	182 (19.1)	
	Obesity	17 (19.8)	69 (80.2)	86 (9)	
Academic Year: N (%)	2^nd^ year	45 (20.2)	178 (79.8)	223 (23.4)	0.543
	3^rd^ year	57 (23.5)	186 (76.5)	243 (25.5)	
	4^th^ year	61 (22.3)	213 (77.7)	274 (28.8)	
	5^th^ year	11 (22.4)	38 (77.6)	49 (5.1)	
	6^th^ year	10 (23.8)	32 (76.2)	42 (4.4)	
	Graduated	36 (29.5)	86 (70.5)	122 (12.8)	
Faculty: N (%)	Health-related	106 (23.9)	337 (76.1)	443 (46.5)	0.565
	Non-health	114 (22.4)	396 (77.6)	510 (53.5)	

Prevalence of GERD and related risk factors and lifestyle

According to the aim of this study, which is to assess the prevalence of GERD, GerdQ questionnaire for the diagnosis of GERD was used. The results revealed that the overall prevalence of GERD among the study participants was found to be 23.1%.

Concerning risk factors related to GERD, as shown in Table 2, most of the participants (50.7%) had no physical activities for 30 minutes or more per week and 26.1% had one to two physical activities per week. The most used type of analgesics was paracetamol which is taken by 50.8% of the participants while 39.5% did not use any analgesics. About the frequency of analgesics use per month; most of the participants (38%) used it 2-5 times per month and (22.5%) used it once or less.

**Table 2:** Risk factors and lifestyle and its association with GERD (n=953) *P-value was calculated using chi-square test. Percentages were calculated within each column. GERD: gastroesophageal reflux disease; NSAID: non-steroidal anti-inflammatory drugs. PPI: proton pump inhibitors.

Variable	GERD		Total N (%)	P-value
	Yes N (%)	No N (%)		
Physical activities ≥ 30 min. / Week				
Never	129 (58.6)	354 (48.3)	483 (50.7)	0.001*
1-2	49 (22.3)	200 (27.3)	249 (26.1)	
3-4	32 (14.5)	86 (11.7)	118 (12.4)	
5 or more	10 (4.5)	93 (12.7)	103 (10.8)	
Most types of analgesics used				
Non	87 (39.5)	289 (39.4)	376 (39.5 )	0.802
NSAID	24 (10.9)	66 (9)	90 (9.4)	
Paracetamol	109 (49.5)	375 (51.2)	484 (50.8)	
Both of them	0 (0)	2 (0.3)	2 (0.2%)	
Others	0 (0)	1 (0.1)	1 (0.1)	
Use of PPI				
Yes	40 (18.2)	28 (3.8)	68 (7.1)	0.000*
Number of meals/day				
< 3 Meals	107 (48.6)	370 (50.5)	477 (50.1)	0.020*
3 Meals	56 (25.5)	232 (31.7)	288 (30.2)	
> 3 Meals	57 (25.9)	131 (17.9)	188 (19.7)	
Most types of foods				
Fatty				
Yes	450 (61.4)	116 (52.7)	566 (59.4)	0.022*
Spicy				
Yes	95 (43.2)	323 (44.1)	418 (43.9)	0.817
Chocolate				
Yes	98 (44.5)	276 (37.7)	374 (39.2)	0.066
Fibers rich food				
Yes	39 (17.7)	202 (27.6)	241 (25.3)	0.003*
Most types of drinks				
Tea				
Yes	77 (35)	271 (37)	348 (36.5)	0.594
Coffee				
Yes	106 (48.2)	397 (54.2)	503 (52.8)	0.119
Soft Drinks				
Yes	108 (49.1)	338 (46.1)	446 (46.8)	0.437
Do you have a habit of midnight meals?				
Yes	124 (56.4)	429 (58.5)	553 (58)	0.569
During the week how frequently do you take breakfasts?				
Never	50 (22.7)	152 (20.7)	202 (21.2)	0.622
3 or less	85 (38.6)	261 (35.6)	346 (36.3)	
> 3	42 (19.1)	161 (22)	203 (21.3)	
Daily	43 (19.5)	159 (21.7)	202 (21.2)	
Do you have a habit of quick eating?				
Yes	76 (34.5)	298 (40.7)	374 (39.2)	0.104
Do you wear tight clothes or compression belts?				
Yes	63 (28.6)	207 (28.2)	270 (28.3)	0.909
Khat chewing				
Yes	13 (5.9)	18 (2.5)	31 (3.3)	0.011*
Smoking				
Yes	22 (10)	40 (5.5)	62 (6.5)	0.056
No	189 (85.9)	663 (90.5)	852 (89.4)	
Ex-smoker	9 (4.1)	30 (4.1)	39 (4.1)	
Family History of GERD				
Yes	37 (16.8)	68 (9.3)	105 (11)	0.000*
No	77 (35)	413 (56.3)	490 (51.4)	
Don’t know	106 (48.2)	252 (34.4)	358 (37.6)	
Medical history of chronic diseases				
Yes	38 (17.3)	91 (12.4)	129 (13.5)	0.065

Only 7.1% of the participants use PPI such as omeprazole, esomeprazole, lansoprazole, pantoprazole, and rabeprazole. Participants were asked about their number of meals per day; most of them (50.1%) have less than three meals. The most selected type of food by the participants was fatty foods (59.4%) followed by spicy foods (43.9%) and the most selected type of drink was coffee (52.8%) followed by soft drinks (46.8%).

Regarding their habits; 553 (58%) of the participants have a habit of midnight meals and 468 (49.1%) have a habit of having dinner within an hour to two hours before bedtime. Also, they were asked about the frequency of taking breakfasts during the week; most of them (36.3%) take breakfast three times or less. Only 3.3% of the participants chewed khat with a frequency of khat chewing per week as follows: less than once (19.4%), 1-5 times (71%), 5-10 times (6.5%), and more than 10 times (3.2%).

Only 6.5% of the participants are current smokers and when they asked about the number of cigarettes they take daily, their answers were as follow: 1 to 10 cigarettes (54.8%), 11 to 20 cigarettes (32.3%), and more than 20 (6.5%) while 4 (6.5%) use electronic cigarettes.

Eleven percent of the participants have a family history of GERD and 13.5% have a medical history of chronic diseases. Regarding their chronic illnesses, 77 had asthma (59.7%), 25 had diabetes mellitus (19.4%), 11 had anemia (8.5%), ten had hypertension (7.8%) and eight had other illnesses (6.2%).

The relationship between GERD and different risk factors related to GERD were assessed. A statistically significant association (p < 0.05) was found with the following factors: physical activity, use of PPI, No. of meals per day, fatty foods, fibers rich foods, dinner before bedtime, khat chewing, and family history of GERD.

Overall, the following characteristic was observed among those who are considered as having a higher prevalence of GERD: have no physical activities ≥ 30 minutes/week (58.6%), consumer of PPI (18.2%), having > three meals per day (25.9%), who do prefer fatty foods (47.3%) and fiber-rich foods (82.3%), who chew khat (5.9%) and participants with a family history of GERD (16.8%). More information about the factors associated with GERD is provided in Table 2.

GERD complications

Regarding GERD complications, the most reported one was sore throat (5.9%) followed by sinusitis and hoarseness (5.5%), chronic cough (3.5%), and asthma (3.1%). Three patients (0.3%) reported having esophageal cancer. Other reported complications are presented in Table 3.

**Table 3:** Distribution of gastroesophageal reflux disease (GERD) complications among the participants

GERD Complications	Frequency	Percent
Erosive esophagitis	16	1.7%
Esophageal stricture	8	0.8%
Abnormal change in the lining of the mucous cells of the esophagus	9	0.9%
Esophageal cancer	3	0.3%
Swollen throat	22	2.3%
Sore throat	56	5.9%
Sinusitis	52	5.5%
Tooth erosion	22	2.3%
Hoarseness	52	5.5%
Throat infection	27	2.8%
Vocal cord granulomas	4	0.4%
Subglottic stenosis	3	0.3%
Throat cancer	1	0.1%
Chronic cough	33	3.5%
Asthma	30	3.1%
Lung cancer	5	0.5%

Predictors of GERD

We conducted multivariate logistic regression with GERD as dependent variable. The results revealed that five or more physical activities for ≥ 30 minutes per week were found to decrease the odds of GERD by 65% compared to no physical activity (odds ratio =.35, P-value = .030). Also, fatty foods and fiber-rich foods were found to decrease the odds of GERD (P-value =.019, .001 respectively).

Having more than three meals per day increase the odds of GERD by 79% compared to having less than three meals (odds ratio = 1.79, P-value = 0.032). Also having a family history of GERD increases the odds of GERD by 180% compared to the absence of a family history of GERD (odds ratio = 2.80, P-value = 0.00). The use of PPI was found to increase the odds of GERD (P-value = .000). Other variables did not have significant effects on the odds of GERD. Further information is provided in Table 4.

**Table 4:** Predictors of GERD among the study participants GERD: gastroesophageal reflux disease; CI.: confidence interval. GPA: grade point average. BMI: body mass index. NSAID: non-steroidal anti-inflammatory drugs. PPI: proton pump inhibitors.

	Odd ratio	95% C.I. for Odd ratio	P-value
		Lower - Upper	
age group (≥ 22 years)	0.95	0.59 – 1.51	.817
Female gender	0.96	0.64 - 1.43	.835
Marital status			.097
Single	1		
Married	1.52	.899 - 2.513	.108
Divorced	5.09	1.06 - 23.95	.041
Widowed	0	0	1.000
Rural Residence	1.05	0.73 - 1.50	.805
Academic Year			.553
2nd Year	1		
3rd Year	1.00	0.60 - 1.67	.990
4th Year	0.79	0.43 - 1.44	.434
5th Year	0.76	0.30 - 1.95	.574
6th Year	0.89	0.34 - 2.33	.805
Graduated	1.38	0.69 - 2.79	.365
Non-Health Faculty	0.77	0.54 - 1.09	.138
GPA of 4.00 or more	0.83	0.56 – 1.24	.368
BMI			.348
underweight	1		
Healthy weight	0.71	0.45 - 1.14	.161
Overweight	0.89	0.50 - 1.59	.697
Obesity	0.57	0.27 - 1.24	.157
Physical activities ≥ 30 minutes/week			.030
Never	1		
1-2	0.77	0.50 - 1.20	.253
3-4	1.18	0.70 – 1.99	.527
> 5	0.35	0.16 - 0.77	.009
Most type of analgesics used			.998
Non	1		
NSAID	1.00	0.54 - 1.83	.993
Paracetamol	1.07	0.72 - 1.59	.733
Both of them	0	0	.999
Others	0	0	1.000
Use of PPI	5.19	2.88 - 9.38	.000
Number of meals/day			.032
< 3 meals	1		
3 meals	0.99	0.65 - 1.51	.966
> 3 meals	1.79	1.12 - 2.85	.014
Most types of foods			
Fatty	1.1	0.42 - 0.93	.019
Spicy	0.90	0.63 - 1.28	.551
Chocolate	1.26	0.87 - 1.83	.220
Fibers rich food	0.48	0.31 - 0.75	.001
Most types of drinks			
Tea	1.08	0.74 - 1.59	.676
Coffee	0.83	0.57 - 1.20	.324
Soft Drinks	0.98	0.67 - 1.44	.908
Midnight meals	1.31	0.85 - 2.01	.226
Frequency of taking breakfasts			.515
Never	1		
3 or less	0.99	0.61 - 1.60	.974
> 3	0.70	0.40 - 1.22	.209
Daily	0.92	0.52 - 1.60	.756
Quick eating	0.89	0.61 - 1.31	.559
Wearing tight clothes or compression belts	1.10	0.73 - 1.64	.652
Khat chewing	1.54	0.59 – 4.00	.381
Smoking			.062
Ex-smoker	1.26	0.52 - 3.07	.609
Smoker	2.46	1.17 - 5.21	.018
Family History of GERD			.000
No	1		
Yes	2.80	1.63 - 4.80	.000
I don't know	1.96	1.31 - 2.93	.001
Presence of medical history of chronic diseases	1.40	0.87 - 2.28	.169

## Discussion

In this descriptive and analytical cross-sectional study, we aimed to assess the prevalence and risk factors of GERD among students at Jazan University. GERD is globally recognized as a major health problem for adults and has a significant impact on the health, economic, and health-related quality of life of patients. In this study, a well-validated GERD score (GerdQ) was used to diagnose GERD as well as its complications [1,9].

In our study, we found that the prevalence of GERD in Jazan University students was 23.1%, which is considered relatively high. A previous study was conducted in the Jazan region showed a higher prevalence than our results, which stated that the proportion of participants who scored >8 on the GerdQ (had GERD) was 32.2% [10]. However, when it comes to the studies among university students, our findings were consistent with other studies were conducted in Shaqra university in 2018-2019 and King Abdulaziz University in 2019, which revealed a comparable percent of the prevalence of GERD among students (23.8%, 25.9% respectively) [11,12]. Further, our results are higher than another study in Chinese college freshmen, the prevalence was 5.1% and it is lower than the study that was conducted in 1114 private-tuition students of Anuradhapura in Sri Lanka; the prevalence was 52% [13,14]. These data suggested that GERD symptoms are a significant health problem among Saudi students. University students are prone to GERD and this may be due to psychological stress and poor diet. In addition, we found that sore throat (5.9%) followed by sinusitis and hoarseness (5.5%) were the most frequent complications of GERD.

Our study found that dietary habits, like the type of food, and the number of meals per day, are significantly associated with GERD. The most commonly reported type of food was greasy foods which are statistically significant to increase odds of GERD. Moreover, more than three meals per day were found to increase the odds of GERD (P-value < 0.05). In a previous study that was conducted in Sri Lanka, it was found that irregular dietary habits were found to be associated with GERD symptoms [13]. In contrast to another study in Shaqra University in Saudi Arabia, the authors reported that types of diet related to fatty and spicy food did not show a significant relation [11]. The most common selected drinks were coffee and soft drinks but without significant association. Regarding physical activity, our results showed that five or more physical activities for ≥ 30 minutes per week were found to decrease the odds of developing GERD (P-value = .033), and this was supported by another study [15]. Regarding non-modifiable risk factors, we found that older age is a statistically significant risk factor, despite the mean age of our participants being 22 ± 2.9 years, but this can be explained by the wide range of age group (range 17 - 54 years). This finding is in agreement with a previous study of risk factors of GERD in Southwestern Saudi Arabia, which revealed the same association [10]. On the other hand, this association is not compatible with other studies in Iran and other parts of Saudi Arabia, in which they found no correlation between age and GERD, most likely due to the younger age group in those studies [11,16]. Furthermore, family history of GERD in this study was found to increase the odds of developing GERD (P-value = 0.001), and this finding is consistent with another study in Saudi Arabia and maybe indicated a genetic factor [12].

As shown in previous results [10,11,16], our results showed a significant association between GERD and smoking. Smoking can reduce esophageal pressure and exacerbate GERD, increasing the prevalence of GERD in current smokers compared to nonsmokers [10]. This is confirmed by our findings, which showed that there is a positive association between smoking and the development of reflux disease. However, other studies revealed no association between GERD and smoking [12,14]. Moreover, Khat chewing is traditionally practiced by different groups in our region, and the use of Khat chewing was a statistically significant risk factor of GERD (p<0.05). This result is reported by another study was conducted in the Jazan region [10], this probably due to the high number of Khat users in this area of Saudi Arabia which made this association prominent. Regarding pharmacological drugs, PPI showed a significant association with GERD. Understandably, a higher proportion of students with GERD used PPI to relieve symptoms. This finding goes in line with the results of another study in India [17].

The current data are unique when it comes from a relatively large population (n = 953) and represent one of the strengths of our research. However, this study bears many limitations; for example, if the questionnaire had been answered through interviews or clinics, this survey could have provided more accurate data. We also asked students to comment on GERD symptoms and current episodes of their lifestyle to minimize recall bias. Thus, we recommend larger prospective studies of GERD risk factors, their treatment, and complications to enhance our understanding of risk factors and use them to eliminate or mitigate GERD complications in this region. In addition, modifiable factors related to GERD, such as poor dietary habits and lack of physical activity in the target population, need to be addressed to raise students' awareness.

## Conclusions

In this study, we report a high prevalence of GERD among university students at Jazan, a prevalence that was previously reported in other studies on university students. Further, a significant association was found between GERD and other factors such as demographic and dietary habits. Thus, the implementation of public health campaigns to raise awareness about the disease and its risk factor is warranted, and studying the association between GERD and related factors on a larger population in our region could help to enhance our understanding of GERD and minimize the burden of disease.
